# Observations Regarding Chronic Heart Disease Complicating Pregnancy and Labour

**Published:** 1894-06-16

**Authors:** J. C. Webster

**Affiliations:** Assistant to the Professor of Midwifery in the University of Edinburgh


### MEDICAL PROGRESS AND HOSPITAL CLINICS.

[*The Editor will be glad to receive offers of co-operation and contributions from members of the profession. All letters should be addressed to* THE EDITOR, THE LODGE, PORCHESTER SQUARE, LONDON, W.]

### OBSERVATIONS REGARDING CHRONIC HEART DISEASE COMPLICATING PREGNANCY AND LABOUR—(*continued*).

By J. C. WEBSTER, M.D., F.R.C.P.Ed., Assistant to the Professor of Midwifery in the University of Edinburgh.

When labour sets in the symptoms vary greatly in different cases in regard to time of onset and intensity. Sometimes the labour may go on exactly as a normal one, the patient not complaining in any way. In other cases there may only be the slightest signs of palpitation or breathlessness. These favourable cases are mostly found in aortic disease. Sometimes the bad symptoms begin in the first stage, sometimes in the second, sometimes in the third, sometimes immediately post-partum, or at a later period in the puerperium.

In the first stage trouble may start, or symptoms already present may become aggravated. There may be palpitation or attacks of dyspnœa. The exacerbation is due to the increased strain on the heart, and to the pulmonary congestion during the periods of uterine contraction when the circulation of blood through the uterus is interfered with. If straining efforts are made by the patient, the symptoms are more marked. At the termination of the first stage after rupture of the membranes, slight relief is experienced in some cases as a result of some diminution in the size of the uterine mass owing to the escape of liquor amnii.

During the progress of the second stage the condition of the patient tends to become worse. This is probably chiefly due to the straining efforts made to bring into action the accessory powers. Moreover, the uterine contractions are longer and more powerful, and there is a resulting greater strain on the heart from the interference with the uterine circulation. The pulse may become more rapid, irregular, or intermittent, the breathing may be rapid and oppressed, the patient being restless, and having a feeling of great anxiety.

As the child escapes there may be a feeling of relief on the part of the patient from the great diminution in abdominal distension, but this may be counterbalanced by another factor, viz., the interference with the circulation caused by the retraction of the uterus which occurs after the birth of the child. The exact condition of the pelvis and its contents at the end of the second stage has not yet been worked out by sectional anatomy, and we do not know to what extent the circulation through the retracted organ is arrested. There can be no doubt that it must be greatly slowed, less blood circulating through it, while more is thrown into the non-uterine vascular system. As a result of this there is an increased amount poured into the right side of the heart. In mitral cases this, of course, increases the patient's risk, and often the symptoms become much worse at this time. Yet it is very rare that the patient dies immediately after the child is born. The most dangerous period is yet to follow.

It is at the end of the third stage that there is the greatest danger in mitral disease, especially in stenosis cases. The placenta may be born all right, but immediately or soon afterwards the patient may die. What is the explanation of this? Let us look at the condition of the pelvis at this time. In my researches[30] into the anatomy of the normal pelvis during the puerperium I found that normally after the delivery of the placenta, the retracted and firmly contracted uterus forms a large rounded mass which fills the pelvis like a ball-plug, compressing all the extra-uterine tissues against the bony wall. As a result of this the circulation in the great mass of the uterus is practically checked, the organ being quite anæmic, but also, owing to the pressure of the uterus, the circulation in the extra-uterine pelvic tissues is greatly interfered with. The only congested parts are the small lower uterine segment, the cervix, the vaginal wall and neighbouring parts of the pelvic floor. It is this very great alteration in the circulation which throws the extra burden on the already over-burdened heart. The whole vascular area of the body has been very greatly diminished, and the extra strain on the right side of the heart may be too much for it; over-distension of its already weak and thinned walls results, and its paralysis may follow. This condition in mitral disease was first pointed out by Spiegelberg. It has recently been described by Berry Hart,[31] and I have also noticed it in a case of my own where death occurred. Fritsch,[32] Barbour,[33] and others deny that death is caused in this way. They think that extra strain is not thrown on the right side of the heart, but believe that the blood thrown out of the uterine circulation is accommodated in the extra-uterine vessels of the pelvis and abdomen, owing to the change in intra-abdominal pressure conditions consequent upon the emptying of the uterus. They say that the condition brought about is one of syncope, and that the cardiac failure results because an insufficient amount of blood reaches the heart, which consequently begins to beat irregularly; this along with the nutritive defects leading to a fatal ending.

It is important clearly to understand the difference between these two views, chiefly because the methods of treatment based upon them differ so markedly. There is no proof of Fritsch's theory. Pathological evidence goes against it. In post-mortem cases there is usually found distension of the right side of the heart, or marked distension especially of one or both auricles, and pulmonary congestion. During life the face does not show signs of syncope, but is flushed or deeply cyanosed during the period of danger. Fritsch's theory cannot explain the death which follows emptying of the uterus in early pregnancy. It is, indeed, very interesting to note that the strain thrown on the heart by an abortion may prove too much for it. Moreover, were the theory correct, syncope might be expected often to follow normal labour, whereas it is very rare, save where much blood has been lost. That some of the uterine and pelvic blood is accommodated in abdominal parietal and visceral vessels after the uterus is empty and contracted, is undoubtedly true. That this does not go on to extent described by Fritsch is equally true. The congestion of these vessels that does occur is really a condition of safety for the patient, for the strain on the heart will be for a time diminished in proportion to the amount of blood accumulated in them. Very soon, however, the vaso-motor mechanism will tend to diminish this accumulation, thereby correspondingly increasing the burden on the heart. These various factors must differ considerably in different women, and it is not therefore difficult to understand why there should be such variations in the clinical phenomena witnessed in a series of cases in which the same cardiac lesion exists.

What, now, is the best line of treatment to be adopted in these cases? As regards the relation of heart disease to marriage, there is a fairly general agreement of opinion as to the advisability of urging women with a heart lesion not to marry, *i.e.*, when their safety alone is taken into consideration. Some, however, would only insist upon this restriction in cases of mitral disease. When the patient falls pregnant she must be most carefully looked after. Her life should be regular and well-ordered. She must be guarded from strain, worry or anxiety of any kind. The diet should be nourishing but easily digested, and the bowels should be carefully regulated. The patient should be allowed to go out in fine weather, taking easy walks or carriage drives. In the late months she must be kept more at rest. As to medicinal treatment, none may be required. These are the rare cases in which the patient has no bad symptoms at all during her pregnancy. In many cases a cardiac stimulant is required. This is indicated as soon as [any signs of heart-failure show themselves, *e.g.*, breathlessness, cough, increasing irregularity and weakness of pulse, changes in the cardiac physical signs, &c. The best stimulant is undoubtedly strophanthus, providing a reliable preparation can be obtained. The special advantages of this drug over digitalis are now so well known that they need not be specified here. Iron may also be necessary.

If abortion or premature labour threatens, what is to be done? It is difficult to speak decidedly in regard to this point. It may be stated positively, however, that in the majority of cases there is no necessity for emptying the uterus, and it must never be forgotten that even early emptying may be as bad for the mother as a full-time labour. Often, however, the opinion of the parents is so strong as to prevent such a course from being carried out even if it be advised. In these threatened cases, the abortion may be tided over, and the dangerous symptoms which have developed be removed if the patient be bled once or twice. If the threatened premature delivery cannot be tided over, the case must be treated on the lines to be laid down for full-time deliveries.

If the pregnant woman suffers greatly is it justifiable to empty the uterus? Here, again, different opinions are expressed. My own opinion is that the quiescent condition of pregnancy is less dangerous to the heart than the disturbance brought about by the emptying of the uterus. Bleeding should be tried once or twice in such a case; but if the patient continued to get worse it is very doubtful if it be better to deliver her artificially, before the heart becomes too weak. Such extreme cases are apt to prove fatal whatever course be adopted.

When labour sets in what is to be done? The patient should be carefully watched from the first. If she has been previously in good condition she may be allowed to go through the first stage without interference, an occasional dose of a stimulant being given if necessary. If she be very restless or makes straining efforts she should be quieted. If it is impossible to manage this, and if signs of heart-failure appear, the patient should be chloroformed and the cervix dilated with Barnes' bags.

We are now at the second stage. In some cases she may pass through this stage without trouble. In bad cases, however, especially in mitral disease, she should not be allowed to pass through it in her own strength. She should be chloroformed by an assistant who gives his whole attention to his duty, while the child is extracted with forceps. Occasional hypodermic injections of ether may be required. I wish, however, especially to recommend the use of nitrite of amyl. This drug was first tried with success in heart disease complicating labour by Fraser Wright,[34] who gave it after the third stage was completed, when his patient was in danger of dying of pulmonary and cardiac congestion. Its action is to lessen the strain on the heart through the dilatation which it causes in the small peripheral vessels throughout the body, either from paralysis of the muscular fibres of the arterioles or of the vaso-motor ganglia in them. Soon after its administration (from 20 to 30 seconds) its effects are seen. The drug is best given by the chloroformist in capsules containing four or five minims, which are broken and held to the patient's nose. It is also useful in opposing the tendency to chloroform syncope; a threatening of this condition may be kept off by a careful anæsthetist. As the child is delivered the nitrate of amyl is of great value in neutralising the increasing strain on the heart due to the additional blood thrown out ot the uterine circulation, as a result of the uterine retraction which follows delivery.

The third stage now follows, that most to be feared. Opinions differ regarding the treatment to be employed here. According to the view of those who hold that the danger is due to the accumulation of blood in the abdominal vessels and the consequent diminished supply to the heart, the loss of blood, even in drops, is very dangerous. According to the view which appears to me to be correct, and which I have advocated, the indication is not to conserve, but to allow the free escape of a certain amount of blood from the body in order to prevent over-distension of the lungs and of the right side of the heart. How can this best be brought about? The patient is still kept under chloroform; ether is given hypodermically from time to time, and the nitrite of amyl as required. The chloroform and the amyl nitrite tend to counteract the contractility of the uterus, and so to delay the separation and expulsion of the placenta. This event must not be allowed to take place naturally, because it is apt to cause too sudden a change in the vascular pressure and to prevent the loss of blood, which it is our chief aim to bring about. Neither should the Credé method of expelling the placenta be used, for the same reason. The most satisfactory procedure is to pass one hand into the uterus, separating the placenta gradually, the other hand being placed on the abdomen against the uterus. As the sinuses are torn through blood escapes, the amount lost being carefully watched. In carrying out this operation the greatest skill, coolness, and judgment are required. As the uterus retracts and contracts following the removal of the placenta, the heart should be carefully watched and another dose of nitrite of amyl given if necessary. If, owing to the amount of chloroform and amyl nitrite administered marked contraction does not occur in the uterus, no alarm should be felt. This condition is better than sudden contraction, because the changes in the circulation are more gradually brought about. The organ can easily be compressed between the hands, and if necessary the hot douche can be used, but the latter agency should not be employed save where there is danger of the loss of too much blood. Neither should ergot be used in these cases, except in the last-mentioned condition, because it opposes the escape of the blood from the uterus, which we desire to a certain extent to encourage. Hitherto it has been recommended by many to bleed the patient from the neck or arm. This, it appears to me, is altogether unnecessary, when we have at our disposal the easy method of bleeding from the uterus that I have just described. I have attended five cases successfully through their labours, carrying out this treatment. Another case, my first, I treated according to the other plan to which I have referred, viz., that in which the loss of blood is prevented; the patient died immediately after the delivery of the placenta. At the post-mortem the lungs and right side of the heart were found to be enormously engorged with blood.

The treatment of heart cases during the puerperium is of the greatest importance. Rest in bed for some weeks is advisable. For a time after delivery stimulants of ether and brandy may be required. Strophanthus or digitalis is to be given cautiously. The most easily digested and nourishing food is to be taken. There should be no straining at stool or in making water. The bowels should be regulated so as to move easily, and for some days the urine should be drawn off. The retrogressive changes which take place in the heart during the puerperium introduce a new element of danger, and therefore the greatest watchfulness must be exercised. Complete quiet and good nursing are imperative. The patient should not lose sleep nor be disturbed in any way. As soon as the stomach is able to bear iron it should be given.

